# Editorial: Satellite Earth Observation for animal health and vector-borne diseases

**DOI:** 10.3389/fvets.2024.1484990

**Published:** 2024-10-02

**Authors:** Carla Ippoliti, Pastor Alfonso, Assaf Anyamba, Beatriz Martínez-López

**Affiliations:** ^1^Experimental Zooprophylactic Institute of Abruzzo and Molise G. Caporale, Teramo, Italy; ^2^National Center for Animal and Plant Health (CENSA), WOAH Collaborating Center for the Reduction of the Risk of Disasters, San José de las Lajas, Mayabeque, Cuba; ^3^Oak Ridge National Laboratory (DOE), Oak Ridge, TN, United States; ^4^Center for Animal Disease Modeling and Surveillance, School of Veterinary Medicine, University of California, Davis, Davis, CA, United States

**Keywords:** Earth Observation, satellite remote sensing, geospatial analysis, vector-borne diseases, environmental drivers, climatic variables, big data, animal and public health

This Research Topic, in Frontiers in Veterinary Science, aims to contribute to the latest scientific advances on the use of Earth Observations (EO) for animal health and vector-borne diseases at multiple scales, and to identify pathways for leveraging big EO data to enhance our understanding of the ecological ecosystems behind these diseases, for the benefit of public and animal health.

Four unique manuscripts were published in the Research Topic, each showcasing different applications of EO data. These include studies on disease occurrences, mosquito habitat, and the implementation of a web platform. These studies emphasize the value of EO which provides large volumes of spatio-temporal data over large geographical areas, capturing reflected radiation from surfaces over broad ranges of the electromagnetic spectrum that are converted to different biophysical quantities. Satellite data are available in near-real-time at different spatial and temporal resolutions gathered by platforms managed by various National Space Agencies and, more recently, by a growing number of private or commercial entities. These organizations are developing progressively complex programs to ensure the continuity of multi-decadal acquisition of geophysical and biophysical parameters. Data from National Space Agencies is mostly available free of charge, upon registration and skills to manage and analyse it. Commercial satellite platforms complement the national EO programmes, with a wide range of paid acquisitions but suffer from *ad-hoc* satellite overpasses. Acquisition of commercial data is mostly driven by demand.

Research Topic studies have reported the use of EO data to characterize climatic and environmental conditions under which disease vectors emerge, propagate and result in various disease outbreaks.

The environment, in its broad sense, is the frame in which vectors proliferate, hosts live and interactions among them are facilitated or hampered. In this Research Topic, environmental data was represented by Coordination of Information on the Environment (CORINE), a European program providing land cover and land use data, or Global Land Cover from European Space Agency (ESA), spectral wavelength combinations as Normalized Difference Vegetation Index (NDVI), Enhanced Vegetation Index (EVI), Modified Normalized Difference Water Index (MNDWI) or directly through naive spectral bands. All these datasets provide land use patterns, vegetation cover and temporal dynamics, forest, cropland, and human settlement, all of which can influence the distribution of vectors, animals, their interactions and diseases. Additionally, the datasets allow the identification of water bodies and related habitats that serve as key elements for arthropod vectors and animals and for water-borne diseases.

Together with the land surface characteristics, climate constitutes the driver of the emergence and spread of new and re-emergent infectious diseases. Climate is defined by elements such as temperatures over weeks, months or seasons, precipitation, and derived indices (e.g., evapotranspiration). The contributions highlight the potential use of WorldClim as a climate database of great utility for research on this topic.

Two studies illustrate the “classical” use of EO, where they are employed as variables associated with disease data. The first study (by Condoleo et al.) focuses on the *Toxoplasma gondii* seroprevalence in slaughtered sheep in Central-Southern Italy. CORINE and Worldclim datasets were used to characterize sampled farms. Results highlighted that environmental and climatic variables can be considered as possible risk factors in the spread of toxoplasmosis in the study area. In a second study (Lima et al.), ESA Global Land Cover and Worldclim datasets were used “to delineate climatic and land use profiles” of Colombian municipalities, exploring their correlation with livestock-related variables. Findings highlighted that four distinct livestock-environmental clusters characterize the livestock productive areas of Colombia, allowing coherent surveillance actions based on regional characteristics.

The third study (by Ippoliti et al.) aimed to predict the distribution of *Culex pipiens*, a vector of many vector-borne diseases, by integrating entomological survey in Central Italy with data from Copernicus Sentinel-2. A datacube of 13 spectral bands around each site were used in three models: a baseline model using single-timepoint EO data, a multitemporal model analyzing sequences of images over 2 months, and a model incorporating spatial and climatic relationships via graph neural networks. The study achieved good performances in predicting temporal mosquito distribution, highlighting the effectiveness of using EO data to anticipate vector population patterns and inform surveillance strategies.

Overall, in the EVE (Environmental data for Veterinary Epidemiology) web system presented by Mazzucato et al., environmental and climatic data (LST, NDVI, EVI, MNDWI, precipitation, CORINE) at different spatio-temporal resolutions are available for studies at local, national and continental scale (Triveneto, Italy, Europe, respectively). The web system provides data in pre-defined formats to veterinary epidemiologists and researchers without specific knowledge in Geographical Information Systems (GIS) or remote sensing, integrating EO-derived products from multiple sensors with *in-situ* data.

All four of these studies demonstrate the importance of having EO data available at various spatial and temporal resolutions and frequencies to accurately characterize habitats at local scales, such as for a vector species (as shown by Ippoliti et al.), entire municipalities (as in Lima et al.), or specific regions (as in Condoleo et al.), by associating them with disease cases or animal populations. Indeed, Mazzucato et al. incorporated multiple data resolutions into their system, recognizing that each study operates at specific scale.

For EO data to be increasingly utilized and to fully realize their effectiveness, it is crucial that they are made easily accessible to epidemiologists, in a ready-to-use format, reducing the technical barriers to entry. Consistent and reliable access is also essential datasets (e.g., the NASA repository link has remained unchanged for 20 years), ensuring that long-term studies can rely on a stable data source.

Furthermore, ongoing developments in cloud computing and big data analytics should further simplify the accessibility and utility of EO data. Integrated with new analysis tools and capabilities, the evolving EO resources will increasingly enhance real-time epidemiological monitoring and the rapid response to emerging public health threats.

Future efforts should focus on a multidisciplinary approach to EO data analysis, integrating insights from various fields to improve our understanding of environmental and biological processes underpining vectors emergence and diseases. By combining remote sensing observations with other disciplines, we can refine metrics, develop novel indices, and better characterize complex interactions within ecosystems. This approach will enhance our ability to manage environmental challenges, predict and monitor emerging threats, and ultimately better inform animal health and public health.

